# Genomic and Physiological Analysis of Carbon Storage in the Verrucomicrobial Methanotroph “*Ca*. Methylacidiphilum Fumariolicum” SolV

**DOI:** 10.3389/fmicb.2012.00345

**Published:** 2012-09-28

**Authors:** Ahmad F. Khadem, Muriel C. F. van Teeseling, Laura van Niftrik, Mike S. M. Jetten, Huub J. M. Op den Camp, Arjan Pol

**Affiliations:** ^1^Department of Microbiology, Institute of Water and Wetland Research, Radboud University NijmegenNijmegen, Netherlands

**Keywords:** *Methylacidiphilum*, methane, Verrucomicrobia, carbon storage, glycogen, survival

## Abstract

“*Candidatus* Methylacidiphilum fumariolicum” SolV is a verrucomicrobial methanotroph that can grow in extremely acidic environments at high temperature. Strain SolV fixes carbon dioxide (CO_2_) via the Calvin–Benson–Bassham cycle with methane as energy source, a trait so far very unusual in methanotrophs. In this study, the ability of “*Ca*. M. fumariolicum” to store carbon was explored by genome analysis, physiological studies, and electron microscopy. When cell cultures were depleted for nitrogen, glycogen storage was clearly observed in cytoplasmic storage vesicles by electron microscopy. After cessation of growth, the dry weight kept increasing and the bacteria were filled up almost entirely by glycogen. This was confirmed by biochemical analysis, which showed that glycogen accumulated to 36% of the total dry weight of the cells. When methane was removed from the culture, this glycogen was consumed within 47 days. During the period of glycogen consumption, the bacteria kept their viability high when compared to bacteria without glycogen (from cultures growing exponentially). The latter bacteria lost viability already after a few days when starved for methane. Analysis of the draft genome of “*Ca*. M. fumariolicum” SolV demonstrated that all known genes for glycogen storage and degradation were present and also transcribed. Phylogenetic analysis of these genes showed that they form a separate cluster with “*Ca*. M. infernorum” V4, and the most closely related other sequences only have an identity of 40%. This study presents the first physiological evidence of glycogen storage in the phylum Verrucomicrobia and indicates that carbon storage is important for survival at times of methane starvation.

## Introduction

The use of methane (CH_4_) as carbon and energy source distinguishes the aerobic methanotrophs as a unique group within the methylotrophs. Aerobic methanotrophs are found within the Proteobacteria, Verrucomicrobia, and the NC10 phylum (Hanson and Hanson, 1996; Op den Camp et al., 2009; Ettwig et al., 2010; Semrau et al., 2010). The verrucomicrobial methanotrophs, for which the genus name “*Methylacidiphilum*” was proposed (Op den Camp et al., 2009), were recently discovered. They were isolated in pure cultures from volcanic regions (Dunfield et al., 2007; Pol et al., 2007; Islam et al., 2008), which may be important natural sources of methane (Castaldi and Tedesco, 2005; Kvenvolden and Rogers, 2005). The verrucomicrobial methanotrophic bacteria are able to grow in these highly acidic and hot conditions and might have an essential role in reducing global methane emissions into the atmosphere.

“*Candidatus* Methylacidiphilum fumariolicum” strain SolV is one of the thermoacidophilic verrucomicrobial methanotrophs and its physiology has been studied in some detail. This microorganism can use ammonium, nitrate, or atmospheric nitrogen as nitrogen source (Pol et al., 2007; Khadem et al., 2010), and fixes carbon dioxide (CO_2_) into biomass via the Calvin–Benson–Bassham cycle, using methane as its energy source (Khadem et al., 2011). The latter is in contrast to proteobacterial methanotrophs that use formaldehyde in the ribulose monophosphate pathway (type I) or the serine pathway (type II) for carbon assimilation (Chistoserdova et al., 2009; Semrau et al., 2010).

This study focuses on the growth response of “*Ca*. M. fumariolicum” during nitrogen depletion. When nitrogen is limited and carbon compounds are in excess, methanotrophs, as many other bacteria (Wanner and Egli, 1990 and reference therein), start to accumulate carbon-rich reserve polymers, such as poly-3-hydroxybutyrate (PHB) or glycogen (Linton and Cripps, 1978; Eshinimaev et al., 2002; Pieja et al., 2011a and reference therein). The cells of “*Ca*. M. fumariolicum” are rod-shaped and have a length of 0.8–2.0 μm and a width of 0.4–0.6 μm (Op den Camp et al., 2009). Electron microscopy demonstrated the presence of intracellular inclusions in all three “*Methylacidiphilum*” strains (Pol et al., 2007; Islam et al., 2008; Op den Camp et al., 2009), which might represent storage material. Genes encoding for PHB synthesis are absent from the genomes of the “*Methylacidiphilum*” strains, as is also the case for type I proteobacterial methanotrophs (Hou et al., 2008; Pieja et al., 2011a; Khadem et al., 2012a). However, based on the draft genome of “*Ca*. M. fumariolicum”, genes encoding for glycogen metabolism are predicted (Khadem et al., 2012a).

This study combines growth experiments, transcriptome analysis, electron microscopy, and biochemical analysis to elucidate the ability of glycogen storage in “*Ca*. M. fumariolicum” SolV.

## Materials and Methods

### Organism and medium composition for growth

“*Ca*. M. fumariolicum” strain SolV used in this study was originally isolated from the Solfatara volcano, near Naples, Italy (Pol et al., 2007). “*Ca*. M. fumariolicum” was grown in the standard medium (pH 2) as described before (Khadem et al., 2010), with 10% (v/v) liquid mud pool extract and 2 mM of ammonium.

### Materials

^13^C-labeled CO_2_ was prepared by injecting a 0.6 M NaH^13^CO_3_ (99% ^13^C) solution into a solution of 1.2 M HCl in a closed 60 ml serum bottle. The headspace was then used as source of ^13^C-labeled CO_2_. ^13^C-labeled CH_4_ (99% atom% ^13^C) was obtained from Sigma-Aldrich.

### Fed-batch cultivation

Cultivation of “*Ca*. M. fumariolicum” SolV was performed in a 10 l fermentor (Applikon, Schiedam, Netherlands). The medium (5 l) contained 1.2 mM ammonium. A gas mixture of (all in v/v); 9.5% methane (CH_4_), 23.9% carbon dioxide (CO_2_), and 66.6% air, was supplied to the fermentor in a continuous flow. The oxygen sensor showed a dissolved oxygen level of 8.8% at the onset of cultivation. The pH of the medium was set with sulfuric acid at pH 2 and remained close to pH 2 during growth. The temperature and agitation speed were set to 55°C and 1000 rpm, respectively.

To determine the dry weight, samples of 10 ml from the culture suspension were filtered through pre-weighed 0.45 μm filters and dried to constant weight in a vacuum oven at 70°C.

### Batch cultivation

Batch incubations were performed in serum bottles containing 5% (v/v) medium. The bottles were sealed with red butyl rubber stoppers (Rubber BV, Hilversum, Netherlands). The headspace contained air as the source of oxygen and CH_4_ and CO_2_ concentrations of 10 and 5% (v/v), respectively. The incubations were performed in duplicate at 55°C with shaking at 180 rpm.

In the experiments where methane was removed from the bottles, the cell suspension was sparged with air for about 5 min and 5% (v/v) CO_2_ was added after sealing.

### Cell extracts

Cells were collected by centrifugation (4000 × *g*, 4°C, 10 min). The cell pellet was washed twice in phosphate-buffer (20 mM, pH 7.1) and resuspended in the same buffer. The suspension, which had a final pH of 6, was passed four times through a French press at 20,000 psi and cell lysis (at least 90%) was confirmed by counting DAPI stained cells with light microscopy. Unbroken cells and cell debris were removed from the resulting crude extract by centrifugation at 12,000 × *g* for 30 min (4°C, Sorvall SS-34 rotor).

### Gas analysis

Methane (CH_4_) was analyzed on a HP 5890 gas chromatograph (Agilent, USA) equipped with a Porapak Q column (1.8 m × 2 mm) and a flame ionization detector. ^12^C and ^13^C-labeled carbon dioxide (CO_2_) were analyzed on an Agilent series 6890 gas chromatograph (Agilent, USA) equipped with a Porapak Q and a Molecular sieve column, coupled to a thermal conductivity detector and mass spectrometer (MS; Agilent 5975C inert MSD; Agilent, USA) as described before (Ettwig et al., 2008). For all gas analyses, 100 μl sample of gas was injected into the gas chromatograph.

### Light microscopy

Cell numbers were determined by counting cells in 30 fields (volume per field 2.5 × 10^−8^ cm^3^) of a hemocytometer slide, using an Axioplan 2 imaging phase contrast microscope (Carl Zeiss B. V.).

### Ammonium and protein analysis

Ammonium concentrations were measured using the *ortho* phthaldialdehyde (OPA) method (Taylor et al., 1974). Protein concentrations were measured using the bicinchoninic acid (BCA) assay as described before (Ettwig et al., 2008).

### Elemental analysis

The cells were harvested by centrifugation, after which the pellet was washed with demineralized water and dried overnight in a vacuum oven at 70°C. The dried material (about 0.4 mg) was analyzed on a Thermo Fisher Scientific EA 1110 CHN element analyzer coupled to a Finnigan DeltaPlus mass spectrometer.

### Glycogen assay

The concentration of glycogen in the crude extracts was determined by a two-step enzymatic assay. To enhance the enzyme accessibility of the glycogen granules, crude extracts were first shaken for 10 min at 30 s^−1^ with glass beads (80–110 μm in diameter) in a Retsch MM 301 ball mill. The bead-beaten crude extracts (in triplicate) were then incubated with amyloglucosidase (35 U/ml crude extract in the case of transition phase I and II cells and 17.5 U/ml crude extract for the exponential phase cells) from *Aspergillus niger* (Sigma-Aldrich) in 0.05 M acetate buffer (pH 4.8) for 4 h at 45°C to convert glycogen into glucose. In the second step, the resulting glucose was quantified by the glucose oxidase kit (Sigma-Aldrich). To correct for the amount of glucose already present in the crude extracts, controls of bead-beaten crude extracts that did not undergo amyloglucosidase incubation were also analyzed.

### Chemical fixation and Epon embedding

“*Ca*. M. fumariolicum” cells were fixed in Karnovsky fixative (2% paraformaldehyde, 2.5% glutaraldehyde, 0.025 mM CaCl_2_, and 0.05 mM MgCl_2_ in 0.08 M sodium cacodylate buffer pH 7.4) at 4°C for a maximum of 17 days. The cells were then resuspended in 0.1 M sodium cacodylate buffer (pH 7.4) for 15 min, followed by a post-fixation for 2 h in 1% OsO_4_ and 1.5% K_4_FeCN_6_ in 0.08 M sodium cacodylate buffer (pH 7.4) on ice in the dark. After washing with MilliQ water, the cells were dehydrated in a graded ethanol series (70–100%). Samples were gradually infiltrated with Epon resin. Polymerization of Epon took place at 60°C for 72 h. Ultrathin sections (60–70 nm) of the Epon-embedded cells were cut with the use of a glass knife in a Leica Ultracut UCT microtome.

Before investigation, the sections were post-stained by incubating the grids on drops of 4% uranyl acetate in MilliQ water (30 min in the dark) and 2 min in Reynolds lead citrate stain (Reynolds, 1963), with MilliQ washing in between and afterward. The sections were then investigated in a TEM 1010, JEOL transmission electron microscope. Fifty cells were used for each analysis.

### Polysaccharide (glycogen) stain

Ultrathin sections of chemically fixed “*Ca*. M. fumariolicum” cells (as described above) were treated with the polysaccharide stain as described previously (van Niftrik et al., 2008). In this method, electron dense silver albumin aggregates indicate the presence of polysaccharide molecules.

### Glycogen metabolism genes

Genes encoding proteins involved in glycogen metabolism were identified in the available draft genome of strain SolV by Blast searches (Khadem et al., 2012a) which also showed amino acid identities to homologous proteins. Representative reference *glgA* sequences, encoding the glycogen synthase, were obtained from GenBank and aligned using the MUSCLE aligner in MEGA 5.0 (Tamura et al., 2011). Phylogenetic trees were calculated using the neighbor-joining method with 1000 bootstraps to infer the evolutionary relationship. Positions containing alignment gaps and missing data were eliminated only in pairwise sequence comparisons (pairwise deletion option). The Dayhoff-matrix based method was used to compute the evolutionary distances. For transcriptome analysis RNA was extracted from exponentially growing cells as described before (Khadem et al., 2011). After synthesis of cDNA, single-end Illumina sequencing was performed and transcription analysis was performed using the RNA-Seq Analysis tool from the CLC Genomic Workbench software (version 5.0, CLC-Bio, Aarhus, Denmark) and values are expressed as reads per Kilobase of exon model per million mapped reads (RPKM; Mortazavi et al., 2008). Raw RNA-seq sequence data are available from the GEO depository under accession number GSE40528.

## Results

### Growth response of “*Ca*. M. fumariolicum” upon nitrogen depletion

**Figure 1:**
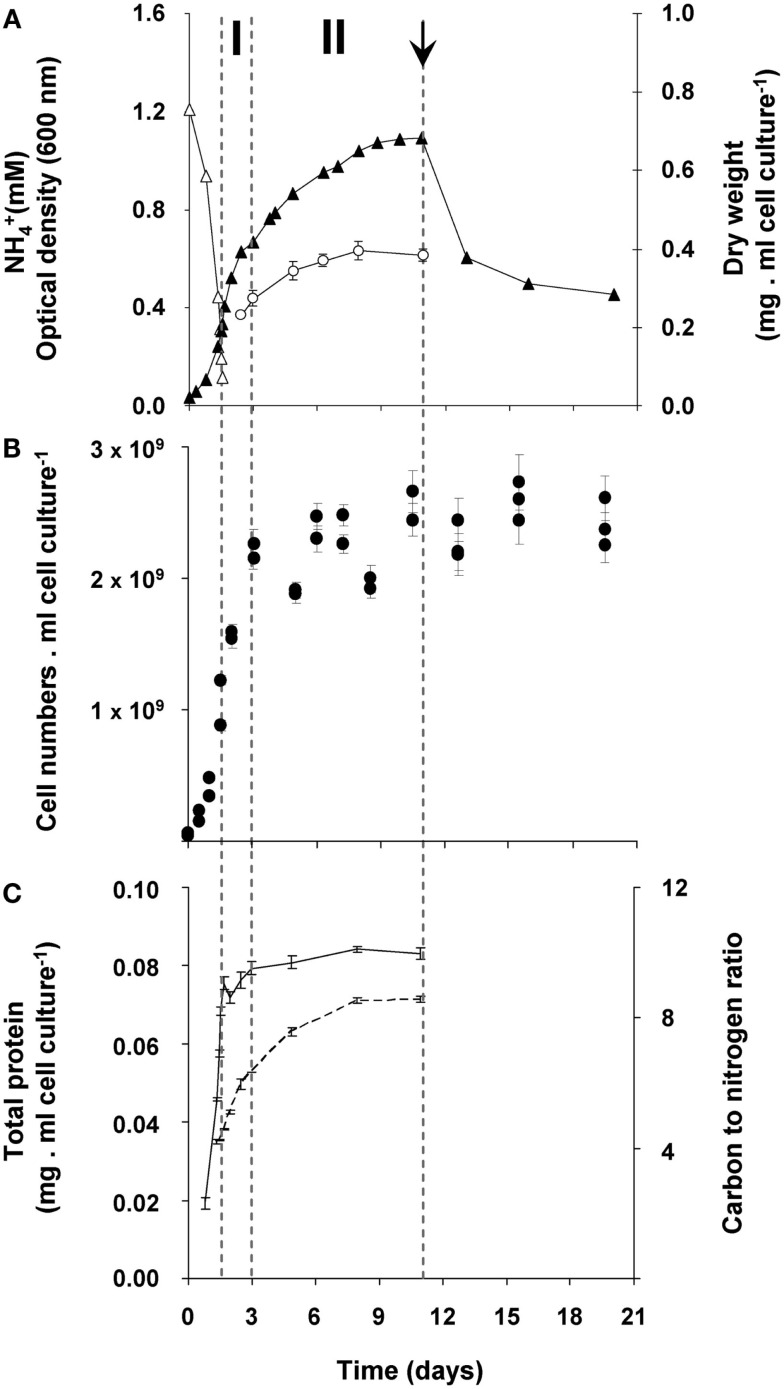
**Growth response of “*Ca*. M. fumariolicum” SolV upon ammonium depletion**. **(A)** Growth was monitored by measuring increase in optical density (closed triangles) and dry weight (open circles). Error bars represent SEM (*n* = 4). Concentration of ammonium is represented by open triangles. After 1.5 days of exponential growth (μmax = 0.07 h^−1^) ammonium was depleted and two phases were observed, indicated by dashed lines and the symbols I and II. At day 11 (arrow), cell cultures were diluted into ammonium containing medium, but without methane added. To compare with the original optical density and cell numbers, values were multiplied by the dilution factor. **(B)** Cell numbers per ml cell culture of “*Ca*. M. fumariolicum” SolV (solid circles). Error bars represent SEM (*n* = 30). **(C)** Total protein (solid line), determined in the crude extracts prepared from harvested cells and carbon to nitrogen ratio of the harvested cells (dashed line). Error bars represent SEM (*n* = 4–5).

In order to study the growth response of “*Ca*. M. fumariolicum” SolV upon nitrogen depletion, the bacteria were cultivated in a fermentor with methane in excess and ammonium as nitrogen source. The dissolved oxygen concentration in the culture was always maintained above 2% O_2_ which prohibits nitrogen fixation to occur (Khadem et al., 2010). After ammonium was depleted, an unexpectedly large increase in optical density was observed that occurred in two phases (Figure 1A). During the first phase (transition phase I) that lasted 1.5 days, both the cell numbers and optical density doubled. At the end of this phase a shoulder in the optical density curve was observed. This seemed to be caused by the fact that growth of the culture stopped, because cell numbers remained more or less constant (Figures 1A,B). However, the optical density still increased for seven more days (transition phase II).

To exclude any possibility of nitrogen fixation following ammonium depletion, the nitrogen in the headspace of the batch incubations was replaced by argon. Before starting the batch incubation, the cultures undergoing exponential growth were washed and put in an ammonium-free medium (methane was present). In these bottles, the same growth pattern was observed as in bottles with nitrogen in the headspace. In addition, growth by uptake of exogenous sources of nitrogen (produced during the exponential phase by “*Ca*. M. fumariolicum”) was excluded. This was done by incubating washed cultures from the exponential phase in medium obtained from a culture at the end of transition phase II. Since no increase in growth parameters was observed in these transition phase II cultures, it could be concluded that all nitrogen in the medium was depleted by the cultures. Again, the same growth pattern was observed as for transition phase I. Moreover, the protein content of the culture remained constant after ammonium depletion (Figure 1C).

**Figure 2:**
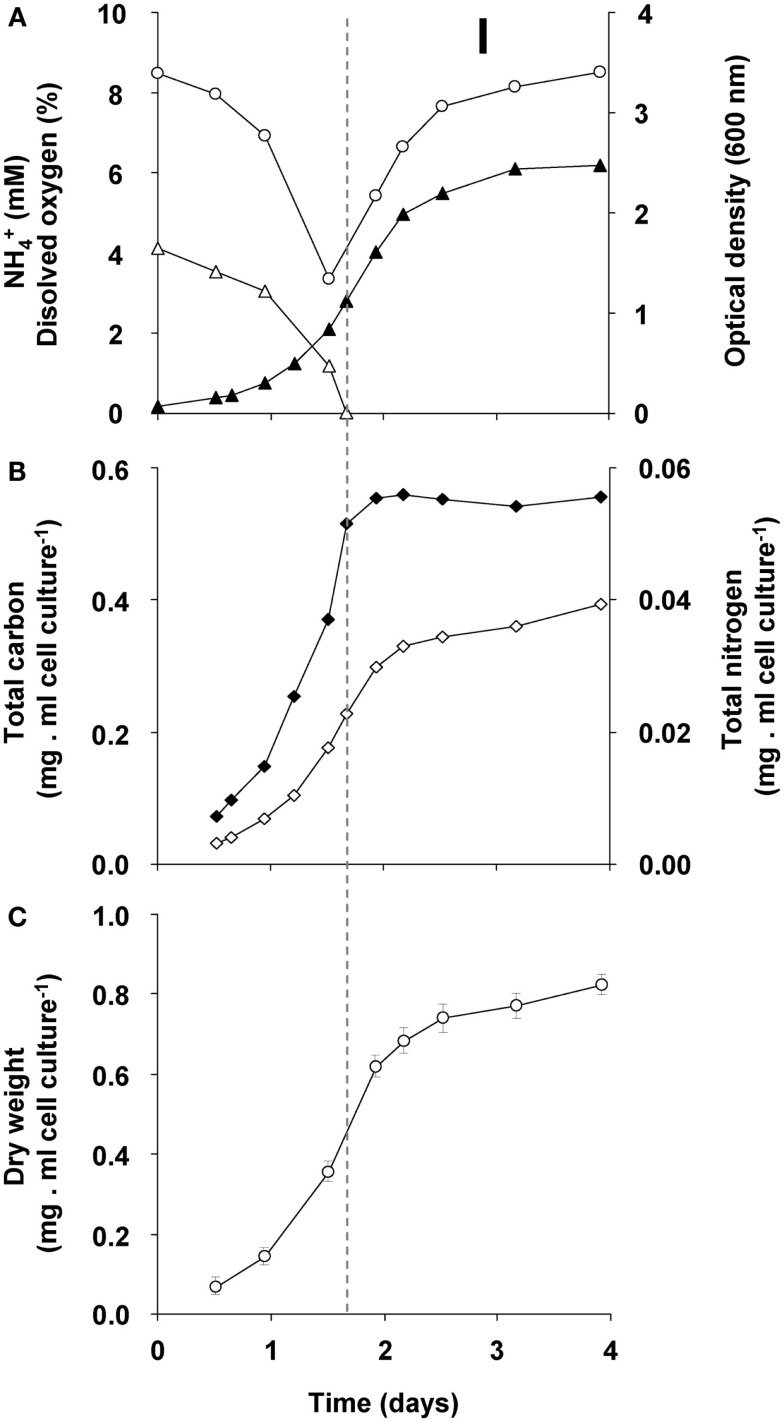
**Growth response of “*Ca*. M. fumariolicum” SolV upon ammonium depletion during transition phase I (the start is indicated by the dashed line)**. **(A)** The optical density (closed triangles), ammonium (open triangles), and dissolved oxygen in culture medium (open circles). **(B)** Total nitrogen (closed squares) and total carbon (open squares). **(C)** Dry weight (open circles). Error bars represent SEM (*n* = 5).

**Figure 3:**
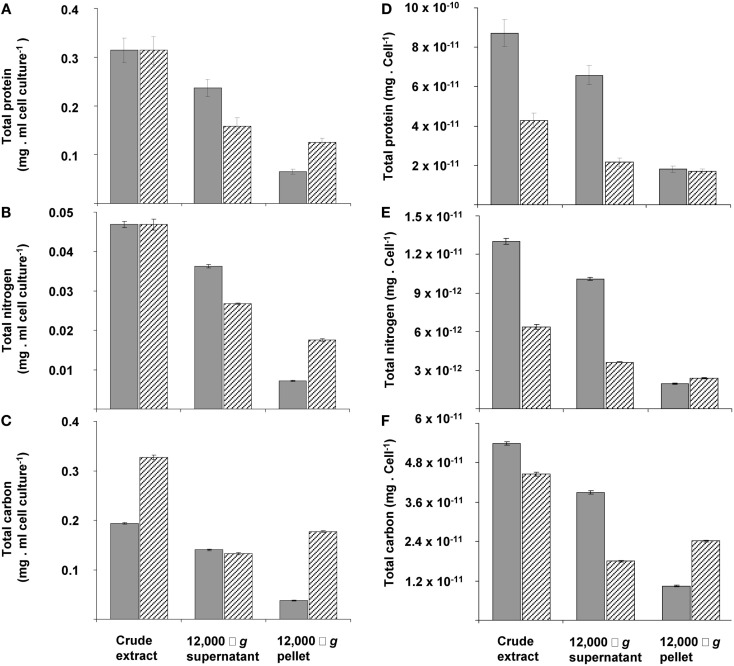
**The distribution calculated per ml of cell culture (A,B,C) or per cell of total protein (D,E,F) of total protein (A,D), nitrogen (B,E), and carbon (C,F) over pellet and cytosol fractions at the beginning (gray bars) and end (dashed bars) of transition phase I in the crude extract, 12,000 × *g* supernatant and 12,000 × *g* pellet**. This experiment was performed with exponentially growing cells that had reached an optical density of 0.5 (see Figure 2A).The amount of total protein and total nitrogen in this exponentially growing cells were normalized to values from cells obtained at the start of transition phase I, since total protein (see Figure 1C) and total nitrogen (see Figure 2B) stabilized after ammonium depletion. The total carbon of the exponentially growing cells was normalized to values from cells obtained at the end of the exponential growth phase (at optical density of 1.12). Error bars represent SEM (*n* = 4).

The changes during transition phase I were studied in more detail in separate cultures with higher cell concentrations (higher initial ammonium concentration). These yielded more accurate dry weight values and allowed preparing adequate quantities of crude cell extracts. Cultures grew exponentially till ammonium was depleted, after which the metabolic rate changed abruptly as indicated by a sharp increase in the dissolved oxygen concentration and the growth rate dropped gradually (Figure 2A). The total nitrogen content of the culture remained constant as inferred from analysis of crude extract of harvested cells (Figure 2B). As cell numbers (Figure 1A), total carbon, and dry weight were all almost doubled during this phase (Figures 2B,C) and cells maintained their normal size, the protein content per cell must have been reduced by half. The consequence of this change of protein content of the cell wall and cytosol was investigated by analyzing the pellet and supernatant fractions of crude extracts of cells at the start (exponentially grown) and the end of transition phase I. When expressed per ml culture, a clear shift of proteins and total nitrogen was observed from the supernatant to the pellet fraction, for which the protein content almost doubled (Figures 3A,B). As the cell numbers in the culture doubled, it means that when results are expressed per cell, the protein and total nitrogen content of the pelleted fraction remained constant at the expense of the supernatant fraction of which the proteins content dropped by a factor of three (Figures 3D,E). In contrast to total nitrogen, total carbon in the pellet fraction increased more than four times for the total culture and more than two times when expressed per cell (Figures 3C,F). This high content of carbon pointed to intracellular particulate storage material.

**Figure 4:**
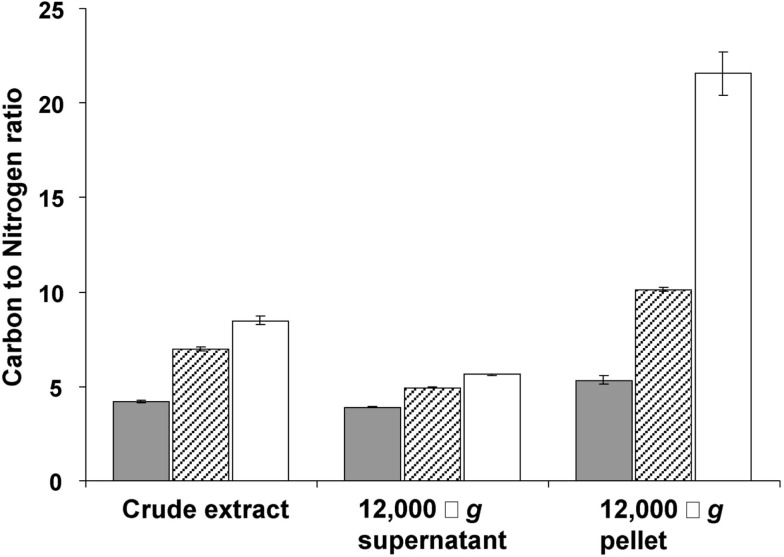
**Carbon to nitrogen ratio (mg/mg) in the crude extract, 12,000 × *g* supernatant and 12,000 × *g* pellet prepared from cells harvested in the exponential phase (gray bars), at the end of transition phase I (dashed bars) and II (white bars)**. Error bars represent SEM (*n* = 3).

This storage seems to continue in transition phase II, where the carbon to nitrogen ratio and dry weight increased at stabilizing cell numbers (Figure 1). Storage of an insoluble form of carbon was most evident from the strong increase in the carbon to nitrogen ratio of the pellet fraction of crude cell extract in both phases (Figure 4).

### Transmission electron microscopic investigation of the cells

**Figure 5:**
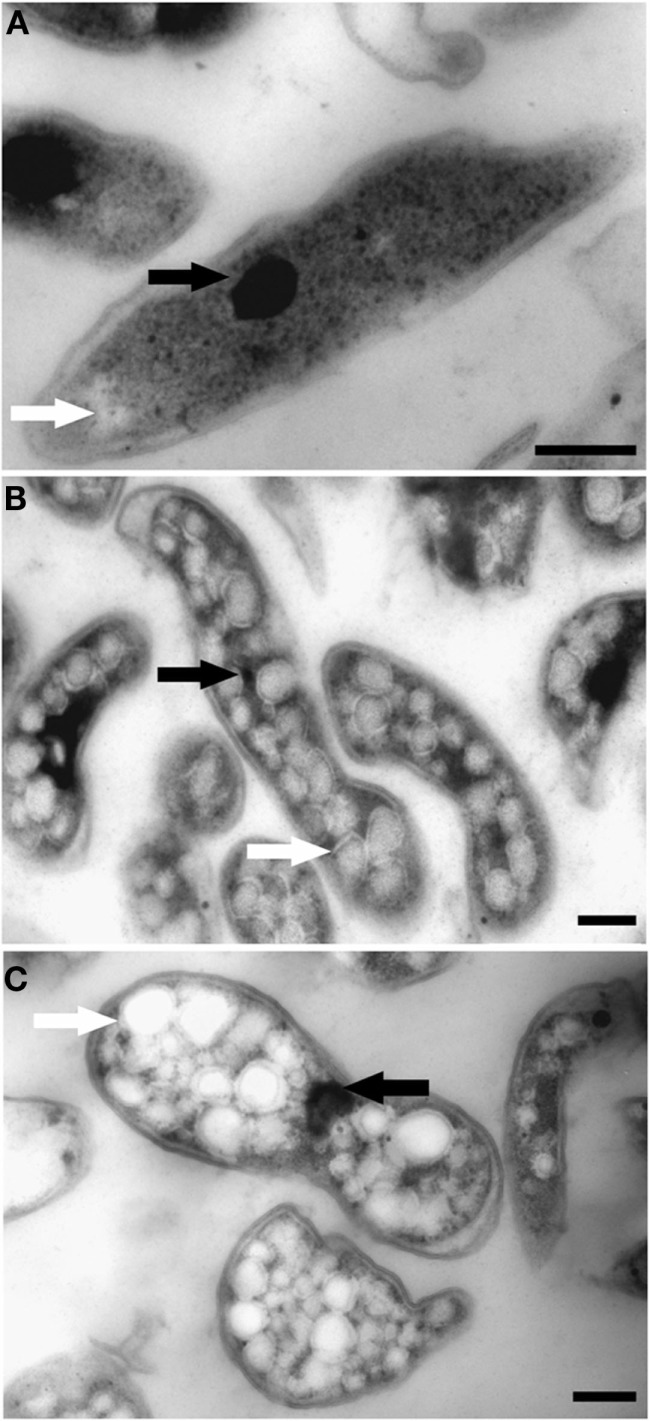
**Transmission electron micrographs showing chemically fixed, Epon-embedded thin sections of “*Ca*. M. fumariolicum” cells in different growth phases**. **(A)** Cell from the exponential phase. **(B)** Cells taken at the end of transition phase I. **(C)** Cells taken at the end of transition phase II. Electron light (white arrows) particles are seen in all growth phases, but are especially abundant in cells of transition phase I and II. Electron dense particles (black arrows) are present in all growth phases. Scale bars, 200 nm.

**Figure 6:**
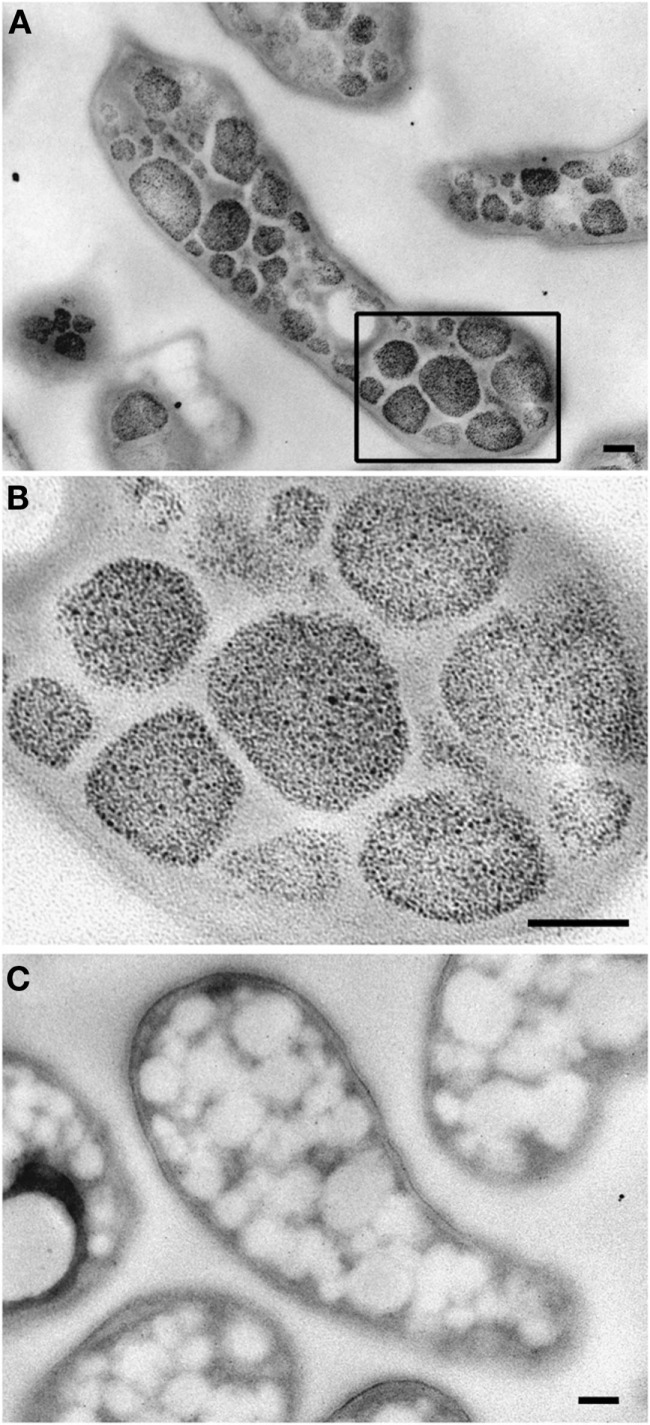
**Transmission electron micrographs showing glycogen staining of chemically fixed, Epon-embedded thin sections of “*Ca*. M. fumariolicum” cells in transition phase II**. **(A)** Glycogen staining is seen in the otherwise electron light particles abundantly present in the cytoplasm. **(B)** Zoom-in of the box drawn in **(A)**. **(C)** Negative control incubated with water instead of periodic acid. Scale bars, 100 nm.

The ultrastructure of “*Ca*. M. fumariolicum” cells from three different growth phases (exponential, end transition phase I and II) was studied by transmission electron microscopy. Circular or ellipsoid (electron light) bodies were observed in high amounts in cells at the end of transition phase I and II (Figure 5, white arrows; Table 1). This resulted in a dense occupation of the whole cell area (as seen in the thin sections) by these bodies. A silver albumin staining performed on cells from transition phase II confirmed that these bodies consist of polysaccharide (Figure 6). In exponentially growing cells these bodies could be discriminated but only in low numbers and smaller in size.

**Table 1:** **Amount and diameter of electron light bodies per cell in different growth phases as observed with transmission electron microscopy of chemically fixed cells**.

Growth phase	Amount of electron light bodies per cell area (1/μm^2^)^a^	Diameter of electron light bodies (nm)^b^
Exponential	8 ± 8	48 ± 15
End transition phase 1	51 ± 19	84 ± 27
End transition phase 2	44 ± 13	96 ± 35

*^a^Analysis performed on 50 cells*.

*^b^Analysis performed on 50 electron light bodies*.

In addition, some “*Ca*. M. fumariolicum” cells featured an elliptical to circular body (100–200 nm in diameter) of high electron density (appeared black in images; Figure 5, black arrows). In most cases only one of these electron dense bodies seemed to be present per “*Ca*. M. fumariolicum” cell, although dividing cells occasionally showed two electron dense bodies.

### Glycogen metabolism genes

In the draft genome of strain SolV (Khadem et al., 2012a) genes encoding for glycogen synthesis (*glgA*, *glgB*, and *glgC*) and degradation (*glgP*, *glgX*, *gdb*, and *pgm*) were present (Table 2). All genes were present in a single copy except for *glgP* encoding the glycogen phosphorylase for which strain SolV possesses three copies. All genes showed orthologs in “*Ca. M*. *infernorum*” V4 (Hou et al., 2008) with amino acid identities ranging from 70–89%. Compared to this, identities to other more distantly related species were always below 47–61%. Phylogenetic analysis of the glycogen synthase (encoded by *glgA*) showed that the verrucomicrobial methanotrophs form a separate cluster. mRNA analysis of cells from an exponentially growing culture by RNA-seq showed transcription of the aforementioned genes comparable to house keeping genes (Table 2 and Khadem et al., 2012b). RNA-seq analysis of nitrogen fixing cells and cells under low oxygen concentration showed comparable transcription levels of glycogen synthesis/degradation genes (Khadem et al., 2012b). Key genes involved in PHB synthesis (*phaC*, *phaA*, *phaB*) are absent.

**Table 2:** **Genes involved in glycogen synthesis and degradation in M. fumariolicum strain SolV and their expression levels**.

Enzyme	Gene name	E.C. number	Accession nr	Identity to V4 ortholog (%)^a^	Expression(RPKM value)^b^
Glycogen synthase	glgA	2.4.1.21	Mfum_1010040	74	306
1,4-alpha-glucan-branching enzyme	glgB	2.4.1.18	Mfum_170041	70	154
Glucose-1-phosphate adenylyltransferase	glgC	2.7.7.27	Mfum_1020013	89	391
Glycogen phosphorylase	glgP	2.4.1.1	Mfum_1020098	81	656
			Mfum_220010	86	113
			Mfum_880004	88	447
Glycogen debranching enzyme	glgX	3.2.1	Mfum_40003	70	381
Amylo-alpha 1,6 glucosidase	gdb	3.2.1.33	Mfum_200059	84	256
Glycosyl transferase (group 1)	rfaG	2.4.1	Mfum_200060	81	43
Phosphoglucomutase	pgm	5.4.4.2	Mfum_550015	73	398

*^a^Comparison with proteins encoded in the genome of *Methylacidiphilum infernorum* V4*.

*^b^mRNA expression in exponentially growing cells as determined by RNA-seq is expressed as RPKM (Mortazavi et al., 2008)*.

### Glycogen assay

To confirm the presence of glycogen as a carbon storage compound in “*Ca*. M. fumariolicum,” a glycogen assay was performed on crude cell extracts prepared from bacteria from an exponentially growing culture and from a culture obtained at the end of transition phase I and II. The glycogen amount as a percentage of dry weight in the crude extracts was 2% for the exponentially growing culture, 26 and 36% for the culture obtained at the end of transition phase I and II, respectively.

### Glycogen consumption and function

**Figure 7:**
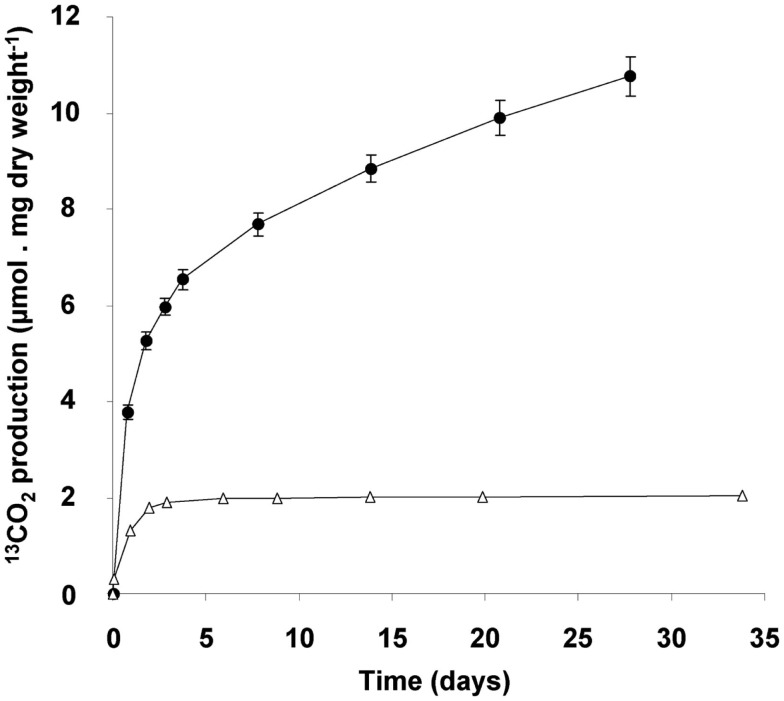
**^13^CO_2_ production originating from ^13^C-glycogen loaded cells (4.4 mg dry weight), obtained at the end of the transition phase II (closed circles)**. Exponentially growing cells (2.1 mg dry weight; open triangles) served as a control. ^13^CO_2_ measured after 1 h of incubation at 55°C, was subtracted from all measured values during the experiment. Error bars represent SEM (*n* = 4).

The consumption of glycogen was detected by the release of ^13^C-labeled CO_2_ from cells that had accumulated ^13^C-labeled glycogen. Accumulation of ^13^C containing glycogen was achieved by growing cultures till late transition phase II in the presence of both ^13^C-labeled methane and carbon dioxide. When such cells are transferred to a medium without methane, stored glycogen is likely to be consumed to meet the energy requirement of the starving cells, and thus ^13^C-labeled CO_2_ is produced. The ratio of ^12^C/^13^C was determined accurately by GC-MS analysis, against a background of 10% unlabeled CO_2_ in the culture bottles. During the first day a rapid ^13^CO_2_ production was observed (initial rate of 5 μmol ^13^CO_2_ produced/mg dry weight of cells/day; Figure 7). This rate dropped gradually to a linear rate of 0.2 μmol ^13^CO_2_ produced/mg dry weight cells/day, which did not change at least till day 28. The total amount of ^13^CO_2_ produced over this period was calculated to be 51.4 ± 1.9 μmol of ^13^CO_2_ (*n* = 4). On basis of the glycogen content of the cells (36% of 4.4 mg dry weight of cells), 9.8 μmol of glycogen was introduced in the incubation. This could result in a maximum of 58.6 μmol of CO_2_ to be produced. This means that about 88% of the ^13^C-labeled glycogen was recovered as ^13^CO_2_. In a parallel experiment it was shown that ^13^CO_2_ production ceased after about 47 days of starvation and during this extended period an additional 9% of the glycogen was converted to ^13^CO_2_ (data not shown). The control incubations with ^13^C-labeled cells from the exponential phase produced only small amounts of ^13^CO_2_, and at lower initial rate (1 μmol ^13^CO_2_ produced/mg dry weight of cells/day), and this production ceased after 3 days. This corresponded with the observation that cells from cultures growing exponentially contained little glycogen (based on both electron microscopy and biochemical analysis).

The possibility of growth on stored glycogen was investigated in a similar experiment as described above without ^13^C-labeling. For this study, cultures that had accumulated glycogen (obtained at the end of transition phase II; Figure 1) were diluted and transferred to a medium without methane but with 2 mM of ammonium in order to allow growth. During 10 days of starvation, in which the ^13^C-labeling experiments suggested that most of the glycogen was already consumed, no growth was observed as cell numbers remained the same (Figure 1A). The optical density decreased rapidly during this incubation (Figure 1B), probably as a result of glycogen consumption. In a parallel experiment in the presence of methane (and no ammonium), the optical density was stable in this period (data not shown). Apparently glycogen in the cell caused some more light scattering; this effect was also observed during the transition phase II, were only glycogen was produced, the optical density increased while cell numbers remained constant (size differences of the bacteria at the different growth phases were only marginal).

**Figure 8:**
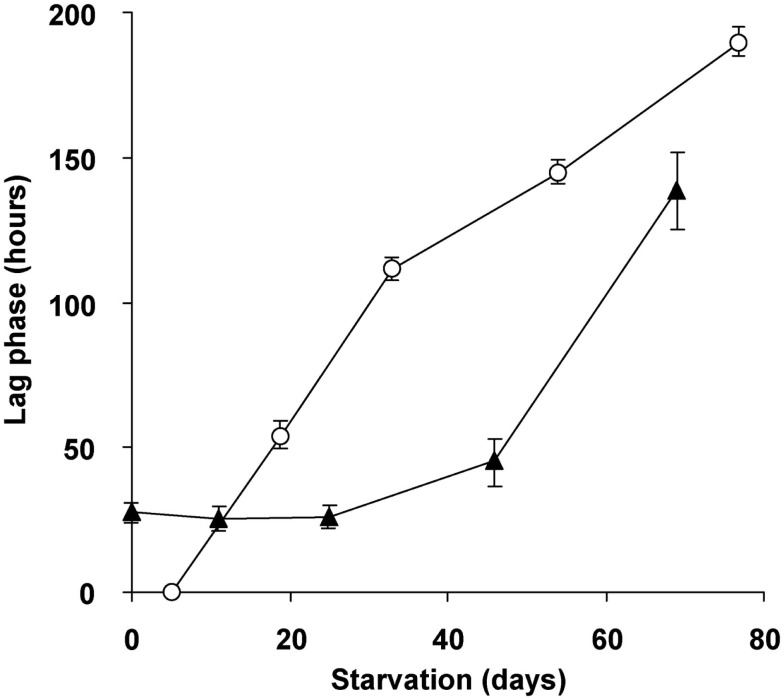
**Viability and recovery of glycogen containing “*Ca*. M. fumariolicum” SolV cells (closed triangles) and exponentially growing cells (open circles) after methane and ammonium starvation for different periods of time**. At time 0, cells were inoculated into optimal medium (4 mM ammonium and methane in the headspace) and lag phases were recorded. Error bars represent SEM (*n* = 3–4).

To study the role of glycogen in viability of the cells, a long-term (70 days) starvation experiment for methane was performed with cultures undergoing exponential growth and cultures that were in transition phase II for 20 days. Exponentially growing cultures were grown till the moment that all ammonium was consumed and the starvation for both cell types was initiated by removing methane from the headspace of the bottles. During the starvation period, cell numbers of both cell types gradually decreased during methane starvation. The cell numbers decreased by a factor of 4.5 in the case of cells obtained from exponentially grown cultures and by a factor 2.4 in the case of cells obtained at the end of transition phase II. The lag phase observed upon recultivation in optimal growth conditions was taken as a measure of viability of the remaining cultures of both types. Exponentially grown cultures were losing viability rapidly after 4 days of methane starvation (Figure 8). Recultivation starting from glycogen loaded cultures showed a lag phase (28 h) from the start. This lag phase remained unchanged for about 40 days of methane starvation (Figure 8). Only after 45 days the lag phase of glycogen loaded cells increased, pointing to a depletion of the stored glycogen (Figure 8). This is well in accordance with the results of ^13^C-labeled glycogen consumption experiment, which showed that in about 47 days all glycogen in the cells was consumed.

## Discussion

Many bacteria store carbon when nitrogen becomes growth limiting (Wanner and Egli, 1990 and references therein). Methanotrophs are also known for their ability to store carbon, either in the form of polyhydroxybutyrate (PHB), glycogen, or by exopolysaccharide (EPS) production. Many studies have focused on PHB storage and its role in methanotrophs, and the genes involved in its synthesis (Pieja et al., 2011b). In addition, EPS production in multiple methanotrophs has been studied in some detail (Malashenko et al., 2001). However, detailed studies about glycogen production and its role in methanotrophs are scarce (Linton and Cripps, 1978; Khmelenina et al., 1999; Eshinimaev et al., 2002).

Although the electron microscopic polysaccharide stain used in our study is not specific for glycogen (Bradbury and Stoward, 1967), our results in combination with the biochemical assay clearly show the presence of glucose polymers in strain SolV. As all the genes for glycogen production and consumption were shown to be present and transcribed, this glucose polymer is most likely glycogen. For more definite proof a biochemical analysis of chain length and type of branching will be necessary.

Based on the growth experiments, it can be assumed that glycogen storage starts soon after ammonium depletion in the medium. However, the glycogen measurements, electron microscopic observations, and the transcriptome data suggest that there was a little bit of glycogen produced during exponential phase. Electron microscopy showed that at the end of the transition phase I (1.5 days after ammonium depletion), cells were packed with glycogen bodies. In addition, the strong increase in total amount of carbon in the pellet fraction per cell, pointed to glycogen storage in phase I.

The increase in dry weight and total carbon (mg/ml cell culture) in transition phase I was not only due to glycogen storage (26% of the dry weight), but also to cell growth, since cell numbers doubled. This growth was not due to other nitrogen sources present in the medium or nitrogen fixation. Nitrogen fixation would also be impossible since dissolved oxygen concentrations in the cultures were too high to support nitrogen fixation by “*Ca*. M. fumariolicum” (Khadem et al., 2010). Moreover, the total protein and nitrogen content (mg/ml cell culture) stabilized immediately after ammonium depletion. The observation that the amount of protein and total nitrogen (mg/ml cell culture) in the pellet fraction of the crude extracts doubled, was in accordance with the doubling of cell numbers.

Completion of cell division once DNA replication has started, seems to be of utmost importance when growth substrates are limiting as has been documented before (Wanner and Egli, 1990 and references therein). While some bacteria become smaller under such conditions (reductive cell division), strain SolV maintained its normal shape and size.

On basis of the carbon percentages, the changes in carbon to nitrogen ratio and assuming a constant amount of nitrogen at the start and end of transition phase II we calculated that dry weight in this phase should have increased about 20%. The measured increase (based on weighing) was about 30%. The increase of the glycogen percentage from 26 to 36% can account for only 15% of the dry weight increase. The observed biomass increase may have resulted from some ongoing growth in this phase, but the increase in cell numbers was too small to result in a significant difference.

Under the circumstances tested (nitrogen depletion), the maximum amount of glycogen measured in the cells of strain SolV was 36% of the dry weight. Under nitrogen limitation, a similar percentage of glycogen was found in the halotolerant methanotroph *Methylobacter alcaliphilus* 20Z using ^1^H-NMR (Khmelenina et al., 1999). Under other growth conditions, percentages of 16% (calcium limitation) and 35% (growth on methanol) are reported for methanotrophs (Linton and Cripps, 1978; Eshinimaev et al., 2002). However, under nitrogen depletion PHB can account for up to 50% of the dry weight in *Methylocystis parvus* OBBP (Pieja et al., 2011b).

Glyceraldehyde-3-phosphate produced from CO_2_ via the Calvin–Benson–Bassham cycle (Khadem et al., 2011), is the most likely precursor of glucose-6-phosphate in strain SolV. The consecutive action of triose-P-isomerase, fructose 1,6-bisphosphate aldolase, fructose 1,6-bisphosphate phosphatase, and glucose-6-P isomerase results in glucose-6-phosphate. The presence of a gene encoding for phosphoglucomutase (*pgm*), suggest that the latter enzyme in turn can convert glucose-6-phosphate and its product glucose-1-phosphate can be used for glycogen synthesis (Wilson et al., 2010). The same route (in reverse) can be used for the degradation of glycogen.

The benefit of accumulated glycogen for “*Ca*. M. fumariolicum” under energy-limiting conditions (no methane) was shown by the fact that cell numbers declined less and that the viability of remaining cells was maintained much better for glycogen loaded cells compared to cells with only small amounts of glycogen (exponentially grown). Glycogen loaded cells showed an initial lag phase which could be expected because the bacteria were in very different metabolic state as a result of the nitrogen depletion, with a much lower cytosolic protein content. This lag phase however became longer only after 40 days of starvation. During this period the glycogen was almost fully consumed, as was shown by ^13^C-labeling experiments. These cells produced ^13^CO_2_ in amounts that equal the initial glycogen content of the cells, while the headspace of exponential cells only showed a small increase in ^13^CO_2_ during the first few days. Growth was only observed in the presence of methane and it could therefore be concluded that glycogen was not used for cell growth. Similar results were found for *M. parvus* OBBP, were no growth on PHB was observed (Pieja et al., 2011b). Therefore, we conclude that the glycogen storage enhances the viability of “*Ca*. M. fumariolicum” during methane starvation.

Regulation of glycogen synthesis/degradation seemed not to be at the transcriptional level since both pathways are simultaneously expressed (Table 2). Having a regulation on protein/enzyme level makes it possible for strain SolV to switch between biosynthesis or degradation of glycogen, depending on the conditions and prevents futile cycling. Bacterial (and plant) glycogen synthases use ADP-glucose as sugar donor and the ADP-glucose availability is the triggering factor for biosynthesis (Cid et al., 2002). Regulation of biosynthesis occurs at the synthesis of ADP-glucose. The enzyme producing this intermediate, glucose-1-phosphate adenylyltransferase (also named ADP-glucose pyrophosphorylase), is allosterically activated about 10- to over 40-fold by glycolytic intermediates and inhibited by AMP, ADP, or Pi (Preiss, 1996). A conserved domain search of the SolV enzyme (Mfum_1020013) amino acid sequence showed all known ligand binding sites to be present. Knowledge on the regulation of the glycogen degradation pathways in bacteria is limiting, but for *Escherichia coli* allosteric regulation of the glycogen phosphorylase by a histidine phosphocarrier protein (HPr) has been described (Seok et al., 2001). The gene encoding this HPr is present in the genome of strain SolV and transcribed under different culture conditions (Khadem et al., 2012a,b).

## Conclusion

The thermoacidophilic verrucomicrobial methanotroph “*Ca*. M. fumariolicum” strain SolV is able to store glycogen in the case of nitrogen depletion. This accumulated glycogen may be consumed in response to energy limitation. It is hypothesized that the bacteria use accumulated glycogen to enhance viability, since growth on accumulated glycogen was not observed. To the best of our knowledge this is the first experimental validation of glycogen storage in the phylum Verrucomicrobia.

## Conflict of Interest Statement

The authors declare that the research was conducted in the absence of any commercial or financial relationships that could be construed as a potential conflict of interest.
